# The Use of Local Anesthesia in Pediatric Dentistry: A Survey of Specialists' Current Practices in Children and Attitudes in Relation to Articaine

**DOI:** 10.1155/2024/2468502

**Published:** 2024-02-12

**Authors:** Thikrayat Bani-Hani, Rami Al-Fodeh, Abedelmalek Tabnjh, Rona Leith

**Affiliations:** ^1^Division of Pediatric Dentistry, Department of Preventive Dentistry, Jordan University of Science and Technology, Irbid, Jordan; ^2^Department of Prosthodontics, Jordan University of Science and Technology, Irbid, Jordan; ^3^Dental Research Unit, Center for Global Health Research, Saveetha Medical College and Hospital, Saveetha Institute of Medical and Technical Sciences, Chennai, Tamil Nadu 600077, India; ^4^Department of Applied Dental Sciences, Faculty of Applied Medical Sciences, Jordan University of Science and Technology, Irbid, Jordan; ^5^Department of Public and Child Dental Health, Dublin Dental University Hospital, Trinity College, University of Dublin, Dublin, Ireland

## Abstract

**Aims:**

This study aimed to evaluate the clinical practices of local anesthesia in children. The study also sought to investigate pediatric dentists' views on articaine infiltration anesthesia and their willingness to use it to replace the inferior dental nerve block in primary molars.

**Materials and Methods:**

A cross-sectional survey was emailed to 183 registered specialists. Descriptive statistics along with chi-square and Fisher's exact tests were used for data analysis.

**Results:**

A total of 72 responses were received. The sample consisted of 62 (86.1%) females and 10 (13.8%) males with varying levels of experience. The vast majority of respondents (98.6%) used topical anesthesia in their practice with children. The most frequently used anesthetic agent was 2% lidocaine (72.2%) followed by 4% articaine (54.2%). The entire sample indicated that they frequently find difficulties in dose calculation for their child patient. Gender and level of experience did not significantly influence specialists' practice or their knowledge of local anesthesia. More than a third (31.9%) of participants were not happy to replace the block anesthesia with articaine infiltration for the treatment of lower primary molars. The most indicated reasons for this unwillingness were lack of effectiveness (11%) and inadequate scientific evidence (11%).

**Conclusion:**

Most pediatric dentists used topical anesthesia with children. Lidocaine was the most commonly used injectable local anesthesia. Specialists' current practices of local anesthesia in children generally conformed well to good standards. However, inadequate knowledge regarding dose calculation was revealed. In addition, specialists' reluctance to use articaine infiltration instead of the block anesthesia was evident in the current population. Further studies, with larger sample size are encouraged.

## 1. Introduction

Local anesthetics are used to reduce or eliminate pain associated with invasive procedures. It is paradoxical that the drug used to eliminate pain can itself be a major source of pain and anxiety. The fear of injection has been reported as the topmost common reason for dental anxiety in children [1]. In order to reduce the anxiety associated with injections, various measures are at disposal for clinicians such as the use of topical anesthetics. In recent years, computer-controlled local anesthetic delivery systems have been proposed for the same purpose, i.e., to reduce the pain associated with injections by controlling the speed of administration and delivering only a small amount of the anesthetic liquid [2].

The administration of local anesthesia in children requires a careful and gentle approach that employs behavior management techniques to encourage child's cooperation. However, these nonpharmacological techniques may not be absolutely effective for all patients, and on some occasions general anesthesia might be required [3].

Clinicians should be aware of and take into account the special considerations for a proper injection technique in children. Failure to appreciate the clinical tips for a painless and successful injection can result in pain and negative experiences with likely life-long dental anxiety [4].

Furthermore, despite their wide use, many dentists are unaware of the important aspects related to the administration of local anesthetics and dose calculation. Kaira and Dabral [5] found that most dentists use local anesthesia routinely without knowing the correct dose calculation. Local anesthesia overdose was observed in 41% of malpractice claims dealing with adverse anesthesia events in pediatric dental patients [6]. This is particularly concerning in children and in patients with special needs such as medically compromised patients. Therefore, it is important that current practices are periodically assessed for adequate knowledge and safety. Thus, the aim of this study was to investigate pediatric dentists' current practices and knowledge of local anesthesia in children.

A secondary aim of this study was to explore pediatric dentists' views in relation to the newly introduced anesthetic agent and injection technique, the articaine infiltration anesthesia, and their willingness to use it to replace the inferior dental (ID) nerve block in pediatric patients. Despite the difficulties and the complications associated with the ID block [7–11], and the reported potential efficacy of articaine infiltration in substituting the ID block in children [12–18], it has been observed that the block technique is still mainly the method-of-choice for anesthetizing mandibular primary molars in Jordan.

The literature is scarce on studies investigating the clinical usage of local anesthesia in pediatric patients [5, 19–22], and no such study has been conducted previously in Jordan. Therefore, the current study aimed to evaluate the clinical practices and knowledge of local anesthesia in children. Secondarily, this study sought to investigate specialists' attitudes regarding the use of articaine infiltration in the treatment of primary molars in children.

The null hypothesis assumed that pediatric dentists' gender, and length of experience had no significant influence on their clinical practices of local anesthesia in children.

## 2. Materials and Methods

### 2.1. Study Design and Setting

This was a cross-sectional survey that sought to explore the current use of local anesthesia in children. The study involved pediatric dentists working in public, private, and academic sectors in various cities in Jordan. It was approved by the Institutional Research Board (IRB reference: 542-2021) committee at the Jordan University of Science and Technology.

### 2.2. Participants and Sample Size

The survey involved all pediatric dentists who were registered at the Jordanian National Society of Pediatric Dentists to avoid any selection bias. All specialists in the various working sectors were approached with the questionnaire with no exclusion criteria.

The sample calculations were performed with the sample size calculator V 2.0 using a single proportion formula based on data from previous relevant research [20, 21]; (proportion (*p*) = 86%, precision = 10%, alpha error = 0.05, power = 0.8). The minimum sample size required was 47. Accounting for 10% potential drop-out, the final study sample was calculated to be 53.

### 2.3. The Questionnaire

A 25-question survey was designed using Google Forms and emailed to all potential participants.

The questionnaire was composed of four sections. Section I included demographic and professional characteristics of participants. Section II explored the use of topical anesthesia. Section III investigated the use of injectable local anesthesia and the clinical technique. The last section (Section IV) sought to capture participants' preference of articaine infiltration or ID block for the various operative and surgical procedures in the lower primary molars in children. It is worth mentioning that in some of the questions, participants were allowed to choose more than one answer.

The questionnaire was generated from some previous surveys in the literature [19–22] and from a series of questions designed by the authors to gather information related to the clinical administration of local anesthesia and injection techniques in children. The questionnaire was initially piloted with five dentists to ensure its clarity. None of the dentists reported confusion or misunderstanding of questions.

Informed consent was obtained from all participants before undertaking the survey. At the start of the survey, the study was introduced to participants (its name, aim, and investigators) along with other information required to consent (voluntary nature, risks, benefits, data confidentiality, etc.). Only participants who consented to take part by selecting the “agree” button were able to move to the next page and get directed to the research questionnaire. All emails were sent between June and October 2022 with follow-up emails sent on two different occasions to remind participants and increase the response rate.

### 2.4. Statistical Analysis

Descriptive analysis of data was carried out using the statistical package IBM SPSS version 25.0. Chi-square test and Fisher's Exact test were used for associations between the various variables. A *p* value of less than 0.05 was considered significant.

## 3. Results

### 3.1. The Study Population

The questionnaire was emailed to 183 registered dentists; however, only 72 responses were received yielding a response rate of (39.3%). The majority of the respondents were females 62 (86.1%) with males accounting for 13.8%. The sample consisted of specialists with varying levels of experience with almost half (47.2%) of them having more than 10 years of experience.  Table 1 shows the demographic and professional characteristics of the study sample.

### 3.2. The Use of Topical Anesthesia

Regarding the use of topical anesthesia, almost all pediatric dentists in the current sample (98.6%) indicated that they used it in children. Fifty-two (72.2%) participants used benzocaine, 17 (23.6%) used xylocaine (5% lidocaine) ointment, and one (1.4%) used EMLA cream (lidocaine and prilocaine).  Table 2 shows the type of topical anesthesia used by participants.

Half of the sample, 36 (50%), applied the topical anesthesia for more than 60 s. The reason cited for not using topical anesthesia was the thought that it was not effective (1.4%). Concerns about anesthetic overdose or patients' acceptance were not indicated by the respondents.

### 3.3. The Use of Injectable Anesthesia and Administration Technique

The most frequently used injectable local anesthetic agent was 2% lidocaine with epinephrine in 72.2% followed by 4% articaine with epinephrine in 54.2% of participants (Table 2).

Regarding the needle length, 34 (47.2%) and 37 (51.4%) of dentists used short and ultrashort needles, respectively, for infiltration anesthesia. For the ID block, 42 (58.3%) used short needles and 29 (40.3%) used long needles in children.

With regards to the administration technique, only 34 (47.2%) put the child in a supine position for the injection. Most dentists (87.5%) indicated that they would always try to hide the needle from the sight of the child.

As for the time required to infuse a complete cartridge, more than 90% of specialists reported that it would usually take them less than 60 s. Just above one-third (33.4%) reported they would “never” or “rarely” need to actively stabilize the child to receive an injection. Most participants (72.2%) were “satisfied” and “very satisfied” with their overall experience with children.  Table 3 summarizes participants' responses (by their gender and length of experience) regarding the clinical administration of local anesthesia in children. The place of practice did not significantly influence the clinical practices of specialists in the current sample.

Concerning the documentation of local anesthesia procedure in patients' records, about one-fifth of participants (18.1%) indicated that they lack a proper documentation system at their practice.

### 3.4. Knowledge of Dose Calculation

The whole surveyed sample indicated that they “always” (19.4%) or “often” (80.6%) find difficulties in calculating the maximum anesthetic dose allowed for their child patient. These difficulties were more statistically significant among the specialists who had more than 5 years of experience compared to the more recently qualified ones (*p* value = 0.008) (Table 3).

The most cited reasons for such difficulties were inadequate dental curriculum (81.9%) followed by the perception that dose calculations were too complicated to understand (62.5%). Lack of training was also reported in 31.9% of dentists.

### 3.5. The Use of ID Block vs. Articaine Infiltration

When asked about injection technique, more than one-third (43.1%) of pediatric dentists indicated that they ‘“always” or “often” use the ID block for treating the lower primary molars in children. A similar percentage of dentists (43.1%) indicated that they “always” or “often” use the “rule of 10” for determining the injection technique (infiltration vs. ID block) when anesthetizing the lower primary molars.  Table 4 shows participants' responses with regard to the factors considered when deciding the injection technique (infiltration vs. ID block) for the lower primary molars.

**Figure 1:**
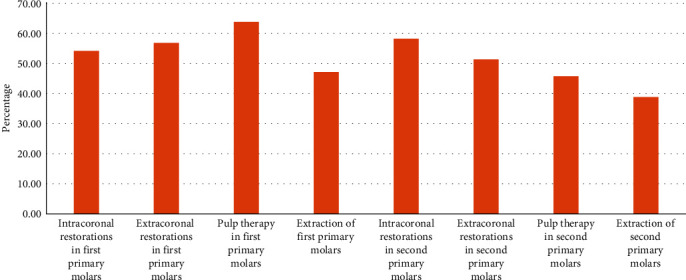
Participants' responses with regard to the use of articaine infiltration anesthesia instead of ID block for the various operative and surgical procedures in lower primary molars.

As for their views on the use of articaine infiltration instead of the ID block in the treatment of lower primary molars, more than half of the participants indicated that they would replace the block anesthesia with articaine infiltration when performing intracoronal restorations (54.2%), extracoronal restorations (56.9%), and pulp therapy (63.9%) in the first primary molars. A lower proportion (47.2%) reported that they would use the articaine infiltration for the extraction of the first primary molars. Almost similar percentages indicated that they would use the articaine infiltration for intracoronal (58.3%) and extracoronal (51.4%) restorations in the second primary molars. However, for pulp therapy and extraction of the second primary molars, only 45.8% and 38.9% of specialists, respectively, were happy to use articaine infiltration instead of the ID block.  Figure 1 shows participants' responses with regard to the use of articaine infiltration anesthesia instead of ID block for the various operative and surgical procedures in the lower primary molars.

It was revealed that 37% of participants rated themselves as “slightly unconfident” and “not confident at all” about using articaine infiltration anesthesia solely for performing pulp therapy or extraction in the second primary molars. Approximately one-third of dentists (31.9%) in the current survey were not happy to replace the ID block with articaine infiltration anesthesia for the treatment of lower primary molars. The most cited reasons for this unwillingness were the lack of effectiveness of articaine infiltration (11.1%) and the inadequate scientific evidence (11.1%). Other reasons such as soft tissue injury (5.5%), systemic toxicity (2.7%), and undesirable side effects (1.4%) were also indicated.

## 4. Discussion

This study investigated pediatric dentists' use and practice of topical and local anesthesia in children in Jordan.

Topical anesthesia was used by almost all specialists in the present sample. This compares to previous reports [19, 20]. Benzocaine was the most used topical anesthetic in the current survey, used by more than two-thirds of the study sample followed by lidocaine gel/ointment. This finding is in agreement with results from Alanazi et al. [22] who reported that benzocaine was the most commonly used kind of topical anesthesia (68.2%) followed by lidocaine (19.3%). Similarly, a survey of pediatric dentists in the United States found that 20% benzocaine topical anesthetic (hurricane) was the most preferred type among respondents [19]. In the literature, several studies compared the clinical efficacy of benzocaine and lidocaine topical anesthetic gels and found that they were equally effective [23, 24]. However, the risk for allergic reactions is increased with the use of esters (benzocaine) topical anesthetics [24]. The European Academy of Pediatric Dentistry (EAPD) best clinical practice guidance [25] emphasizes that clinicians should be aware of the composition and pharmacological properties of each drug or formulation they use.

With regards to the duration of topical anesthesia application, half of the dentists (50%) applied the topical anesthesia for more than 60 s. In the literature, there is a lot of debate regarding the wait time for topical anesthesia. Malamed [24] recommended that topical anesthetics remain in contact with the tissues for 60 s or longer for maximum efficacy.

Among the dentists who never used topical anesthesia in children, the indicated reason was the perception that it was not effective. Previous research by Kohli et al. [19] and Dhindsa et al. [21] reported that 1% and 2% of respondents, respectively, perceived topical anesthesia to be ineffective. The lack of effectiveness can be attributed to the different factors that govern the clinical efficacy of topical anesthetics such as anesthetic's type, formulation, and concentration used, as well as the duration and site of application [26]. However, the evidence generally suggests that topical anesthetics are effective in reducing pain associated with the needle insertion [23, 27–29].

Regarding the needle length, participants used short (47.2%) and ultrashort (51.4%) needles, respectively, for infiltration anesthesia. For the ID block, more than half of the sample (58.3%) used short needles and the remainder used long needles in children. A study involving Saudi dental practitioners found that 93.4% and 3.6% used short and ultrashort needles, respectively, for infiltration. In comparison to the current investigation, the latter survey reported a higher percentage of participants (83.2%) used a long needle for the ID block [20]; however, the Saudi study was not only carried out on specialists but also general dentists which could explain the increased use of long needles in children. In another survey in the United States, short needles were the most frequently used type for infiltrations (84%) and for ID blocks (78%) in children [19]. According to Malamed [24], due to a smaller skull size in children and reduced tissue thickness compared to adults, a decreased depth of injection is required and short needles are usually adequate for block anesthesia. Clinicians treating children should be aware of such anatomical differences and technique variations.

Concerning the clinical administration of local anesthesia, only 34 (47.2%) of specialists in the current sample put the child in a supine position for the injection. A supine position for injection is essential in children not only to prevent syncope reactions in anxious patients [24] but also to aid in hiding the needle from the patient's line of sight to maintain a positive behavior and attitude.

As for the time taken to deliver a complete carpule of local anesthesia, 50% of respondents in the current survey estimated a time of more than 60 s which generally reflects a slow and gentle injection. Malamed [24] recommended at least 60 s for depositing a full cartridge. This rate of deposition ensures little, if any, tissue damage and subsequently less pain “hurt” of injection by patients. Furthermore, a slow injection does not result in serious reactions in case of inadvertent intravascular injection.

Regarding the documentation of the local anesthesia procedure in patients' records, disappointedly, in the current study, about one-fifth of participants (18.1%) indicated that they lack a proper documentation system at their practice. The importance of properly maintained dental records cannot be overemphasized. The American Academy of Pediatric Dentistry [30] recommended that the anesthetic agent, dosage administered, injection technique along with patient's reaction, and/or any complications are all recorded. Dentists are highly encouraged, and in fact, they are ethically responsible, to keep good and complete records for their patients.

Surprisingly, the entire surveyed sample indicated that they “always” or “often” find difficulties in calculating the maximum anesthetic dose allowed for their child patient. These difficulties were more significantly reported among the specialists who had more than 5 years of experience compared to the more recently qualified ones. The latter finding could be explained by the assumption that the more recently qualified practitioners' knowledge might be still fresh to recall these calculations. The concern that many dentists routinely use local anesthesia without being aware of the safe dose and drug calculation has been raised in previous research [5]. The most cited reason for dose calculation difficulties was the inadequacy of the dental curriculum. Henceforth, it is imperative that undergraduate programs and training are assessed regularly to address such deficiencies. In addition, continuous educational courses should be provided to practitioners to ensure safe and professional patient care.

Regarding the injectable anesthetic agents, lidocaine with epinephrine was the most commonly used anesthetic solution in the current investigation, used by almost two-thirds (72.2%) of the study sample, followed by articaine in about half (54.2%) of the respondents. This concurs with previous research [19, 22, 31, 32]. Lidocaine is considered the “gold standard” anesthetic agent with proven efficacy and safety [8]. Several studies compared the clinical efficacy of articaine to lidocaine and reported comparable efficacy [33–35]. In a recent systematic review, articaine was found as clinically effective as lidocaine in all routine dental procedures in patients of all ages [36]. However, according to the EAPD guidance [25] and the original manufacturers' instructions, articaine is not approved for usage in children younger than 4 years of age due to a lack of clinical studies in this age group. Nonetheless, a recent randomized controlled trial included 184 children and found that articaine was safe and effective in children aged 3–4 years [37]. Further research is still required in this regard.

When asked about injection techniques, the overuse of the ID block technique was evident in participants' responses. Despite providing profound anesthesia, the ID block technique has been shown to be more painful than infiltration anesthesia [7, 8]. Furthermore, the ID block has been associated with clinical failures, in about 10%–20% of the cases, which could be attributed to anatomical variations [9]. Other complications such as inferior alveolar and lingual nerve damage [10], and the risk for needle breakage [11] have been reported. In addition, the ID block could be risky in certain subjects like patients with hemophilia or bleeding disorders [10]. The overuse of ID blocks in this study could be partly explained by respondents' over-reliance on the anecdotal guide “rule of 10.” It is worth mentioning this rule, which relies on the tooth to be treated and the patient's age, may be used as a simple guide and it is not a rigid rule. There are many other factors that should be considered in determining the anesthetic technique such as the patient's medical background, the dental procedure, root length, and the surrounding bone.

In children, the lower primary molars may be adequately anesthetized via infiltration technique rather than ID block due to the thin cortical plate and more porous bone [12]. Moreover, with the advent of articaine, which has the ability to diffuse through bone, recent evidence has shown that buccal infiltration with articaine can be a successful and effective alternative to the ID block in children [10, 13–18, 36]. On the other hand, the EAPD guidance [25] states that there is no particular injection technique that is more effective in pain control than others; however, there is probably a benefit from using the ID block for the treatment of second primary molars. Despite the limited evidence, dentists have to be made aware of the potential of articaine infiltration in substituting the ID block, particularly in children. In this survey, more than one-third of participants reported a lack of confidence in using articaine infiltration solely for performing pulp therapy or extraction in second primary molars. No statistically significant difference in relation to articaine use was found among specialists with different levels of experience. This finding contradicts a previous study that reported articaine was more frequently used by the newly qualified dentists [31] and attributed that to the speculation that recently qualified practitioners could be more updated with the recent evidence regarding articaine use. However, in the current survey, this finding could not be confirmed possibly due to the small sample that precluded detection of statistical differences. Similarly, the place of practice did not significantly influence the clinical practices of specialists likely due to the same explanation (i.e., the small sample size).

Overall, no statistically significant differences were detected in the clinical practices of participants according to their gender, or length of experience, thus, we fail to reject the null hypothesis of this study.

It is worth mentioning that the current survey is limited by its small sample. The findings of this survey are not simply generalizable. A larger sized survey with an equal distribution of specialists from both genders in the various groups is recommended in future research. Other limitations include the relatively low response rate, thus, the practices indicated herein do not reflect the entire specialist workforce in Jordan. Furthermore, the current results cannot be extrapolated to the general dental community whose practice with children is likely different and worth addressing in future research.

## 5. Conclusion

Most pediatric dentists use topical anesthesia with children. Lidocaine continues to be the most commonly used injectable local anesthesia. The study obtained valuable insight into the current practices of local anesthesia in children which generally conformed well to good standards. However, it has also revealed inadequate knowledge among pediatric dentists regarding dose calculation.

The recent evidence regarding the potential of articaine to replace the block anesthesia should be considered in practice. Clinicians must strive to know and apply the most up-to-date evidence for local anesthesia in pediatric patients to ensure safe and professional care.

## Figures and Tables

**Table 1:** Demographic and professional characteristics of the study sample.

	Pediatric dentists (%)
Gender	*N*
Males	10 (13.9)
Females	62 (86.1)
Total	72 (100)
Place of current practice	
Public or military service	27 (37.5)
Private clinic	33 (45.8)
Academic institute	12 (16.7)
Years of practice as a specialist	
<2	4 (5.6)
2–5	18 (25.0)
6–10	15 (20.8)
>10	34 (47.2)

**Table 2:** Types of local anesthetics used.

Topical anesthetic	Frequency (%)	Injectable anesthesia	Frequency (%)
Benzocaine	52 (72.2)	2% lidocaine with epinephrine	52 (72.2)
5% lidocaine (xylocaine ointment)	17 (23.6)	4% articaine with epinephrine	39 (54.2)
EMLA cream	1 (1.4)	Plain 2% lidocaine	3 (4.2)
Not sure about the type	1 (1.4)	Others	1 (1.4)
Not using topical anesthetics	1 (1.4)	Not sure about the type	0 (0)

**Table 3:** Participants' responses regarding the clinical administration of local anesthesia in children.

		By gender	By years of experience
		*p* Value^a^	*p* Value^a^
Do you use topical anesthesia in your practice with children?			
Yes	71 (98.6%)	0.073	0.394
No	1 (1.4%)		
How long do you wait for topical anesthesia before you infect?			
<10 s	2 (2.8%)	0.865	0.165
10–30 s	12 (16.7%)		
30–60 s	21 (29.2%)		
>60 s	36 (50.0%)		
How do you position your child patient when administering/injecting local anesthesia?			
Supine	34 (47.2%)	0.195	0.543
Upright	5 (6.9%)		
Semiupright	20 (27.8%)		
Depending on pt.'s cooperation/preference	13 (18.1%)		
Do you try to hide the needle from the sight of your patient?			
Always	63 (87.5%)	0.370	0.394
Often	6 (8.3%)		
Sometimes	3 (4.2%)		
Rarely	0 (0%)		
Never	0 (0%)		
What length of needle do you use most often for infiltration anesthesia?			
Long	1 (1.4%)	0.053	0.813
Short	34 (47.2%)		
Ultrashort	37 (51.4%)		
What length of needle do you use most often for ID block anesthesia?			
Long	29 (40.3%)	0.207	0.058
Short	42 (58.3%)		
Ultrashort	1 (1.4%)		
How often do you get disruptive behaviors associated with injections?			
Always	8 (11.1%)	0.354	0.587
Often	21 (29.2%)		
Sometimes	36 (50.0%)		
Rarely	7 (9.7%)		
Never	0 (0%)		
How long does it take you to inject a full carpule?			
<10 s	5 (6.9%)	0.060	0.544
10–30 s	25 (34.7%)		
30–60 s	34 (47.2%)		
>60 s	8 (11.1%)		
How often do you find difficulties in calculating the maximum dose allowed for your child patient?			
Always	14 (19.4%)	0.363	0.008 ^*∗*^
Often	58 (80.6%)		
Sometimes	0 (0%)		
Rarely	0 (0%)		
Never	0 (0%)		
How often do you get to actively stablize the child with parental asssistance and/or nursing staff to recieve an injection?			
Always	6 (4.5%)	0.339	0.407
Often	31 (23.5%)		
Sometimes	56 (42.4%)		
Rarely	32 (24.2%)		
Never	7 (5.3%)		

*Abbreviations. n*, number; s, seconds; pt., patient. ^a^Fisher's exact test;  ^*∗*^a statistically significant difference.

**Table 4:** Factors considered when determining the type of local anesthesia technique (infiltration or ID block) in lower primary molars.

Factor	Percentage
The age of the patient	62 (86.1)
The tooth being treated	53 (73.6)
The procedure being done	50 (69.4)
Patient's medical background	28 (38.9)
The root length	18 (25)
Surrounding bone	17 (23.6)
Others	5 (6.9)

## Data Availability

Data will be made available upon request by contacting the corresponding author.
